# News and Notes

**Published:** 1903-06

**Authors:** 


### NEWS AND NOTES.

SAXONY has 945 charlatans and 1957 regular physicians.

THE Seventh National Congress of Otology will be held at Bordeaux August 1 to 4.

THE city of Mazatlan, Mexico, has had no new cases of plague for some time, and business has resumed its usual aspect.

THE honorary degree of Doctor of Science is to be conferred by the University of London upon Lord Lister on June 24 next.

THE United States Public Health and Marine Hospital Service has established a hospital at Cape Nome, Alaska, and the hospital at Dutch Harbor will be discontinued.

THE Samuel D. Gross prize of $1,200 will be awarded January 1, 1904. This prize is awarded every five years to the writer of the best original essay on surgical pathology or surgical practice founded upon original investigation. Candidates must be American citizens.

THE fifth meeting of the International Congress of Dermatology will be held in Berlin, September 13 to 17, 1904. The following questions are proposed for discussion: (1) Syphilitic diseases of the circulatory apparatus; (2) skin affections in anomalies of metabolism; (3) epitheliomata and their treatment.

EMPEROR WILLIAM has caused a piece of bark twenty inches in length, bearing the inscription, "His Majesty William II. used this as a splint March 27, in setting the empress's broken arm," to be placed in the Hohenzollern museum. It is now in the William II. room, in a glass case, among gold and silver souvenirs.

---

IN the parish of Acadia, Louisiana, some 225 head of cattle have died recently from charbon. The farmers, ignorant of the infectious nature of the disease, skinned some of the dead animals and sold their skins. As a result thirteen human beings contracted charbon. The police jury of Acadia has been called together to take steps to stamp out the disease.—*Exchange.*

---

THE following statistics have been issued by the Imperial Health Board of Berlin, in regard to the mortality from pulmonary tuberculosis in Europe: Russia has more than 4,000 deaths per million; Austria-Hungary and France, more than 3,000 per million; Sweden, Germany and Switzerland, more than 2,000 per million; Netherlands, Italy, Belgium, Norway, England, more than 1,000 deaths per million.

---

QUEEN WILHELMINA, patroness and founder of the incubator institute, at Amsterdam, has been sued for 2,500 guidens by Francis Gerhard because the latter's boy baby was exchanged for a girl while in the oven. The child substituted is lusty and good-looking, but the parents want their own and say it will be a good thing for the institute if the queen is made to pay, because the officials will then become more careful.

---

The bill which during the early part of the winter was introduced in the Pennsylvania Legislature to legalize the practice of osteopathy and to provide a board of examiners for this so-called school of medicine has, according to *American Medicine,* been defeated. There were twenty-four physicians and two druggists among the members of the House, and it is said that they contributed effectually to the defeat of the measure.

THE ringing of the many church bells of Manila, at all hours of the day and night, with the many "fiesta" days, which seem to be largely celebrated by bell-ringing, has caused the passing of an act by the commission for the abatement of loud and unusual noises. Hereafter the jangling of bells at unseemly hours in Manila, which has been a source of great annoyance to the nervous and those desiring to sleep, will be stopped. The bell-ringing for the early mass, which is celebrated before daylight, has been a special cause of complaint from foreigners.—*Medical Record.*

---

DR. JAMES MORRELL, a young negro physician, went to Elwood, Ind., to settle permanently in that city to practice his profession. He had learned that it was a place of 15,000 inhabitants and expected to find a fair representation of his race in the city. The surprise of the visitor was unbounded when told that there was not a negro in Elwood and that the coming of the negro race had always been discouraged. Dr. Morrell said that he had traveled considerably and did not believe there was another city in the United States as large as Elwood that could say there was not a negro within the corporate limits.

---

OWING to the number of cases of hydrophobia which have occurred in New York within the past few years, it is proposed that a new system, or a more efficient manner of carrying out the present law, shall be initiated in the city. The duty of keeping the streets free from stray dogs lies with the Society for the Prevention of Cruelty to Animals, and it is asserted, says the *Medical Record*, that the society is lax in fulfilling this function. There is no motive to look after ownerless dogs, as no fine can be inflicted, and the society, in the case of the owner of a stray dog without a license caught by its agents being fined, receives a portion of the fine. All stray dogs should be carefully looked after, as such animals are the chief means of spreading rabies.

IN a comparative table of stature, arranged according to nationalities, the United States Indian, says *American Medicine,* stands higher than any other race of the world, though the Patagonian runs him very close. The white citizen comes next. The United States negro ranks fourteenth in the scale, and of all the countries in the world considered the Portuguese are found to be the shortest. It has always been proverbial among anatomists that blond nations are greater than their darker neighbors. This is due to the geologic positions of the blond races. They are characteristic of the north, and on account of the lower degree of temperature are induced to take more exercise, which throws them more in the open air. At the top of the list of countries, arranged in order of stature, the first seven, after the United States white men, are Norway, Scotland, British America, Sweden, Ireland, Denmark, and Holland, all northern nations.

---

IN the course of a lecture to the Conference of Musicians in Dublin, Ireland, some interesting particulars and some astonishing statistics were given relative to the amount of work accomplished by the brain and nerves in piano playing, says the Scientific American. A pianist, in view of the present state of piano-forte playing, has to cultivate the eye to see about 1,500 signs in one minute, the fingers to make about 2,000 movements, and the brain to receive and understand separately the 1,500 signs while it issues 2,000 orders. In playing Weber's "Moto perpetuo," a pianist has to read 4,541 notes in a little under forty minutes. This is about nineteen per second; but the eye can receive only about ten consecutive impressions per second, so that it is evident that in very rapid music a player does not see every note singly, but in groups, probably a bar or more at one vision. In Chopin's "Etude in E Minor" (in the second set) the speed of reading is still greater, since it is necessary to read 3,950 signs in two minutes and a half, which is equivalent to about twenty-six notes per second.

